# From Limited Samples to Mechanistic Insights: Exploratory Identification and Functional Validation of a hsa_circ_0062400/hsa_circ_0002397-miR-338-3p-NRP1 Axis in Myasthenia Gravis

**DOI:** 10.2174/0118715303446341260204093655

**Published:** 2026-03-18

**Authors:** Zhimin Chen, Xiaotong Kong, Ping He, Risu Na, Lihua Wang

**Affiliations:** 1 Department of Neurology, The Second Affiliated Hospital, Harbin Medical University, Harbin, 150086, China;; 2 Department of Neurology, The First Hospital of Harbin, Harbin, 150010, China;; 3 Department of Geriatric Medicine, Tongliao People's Hospital, Tongliao, 020800, China

**Keywords:** Myasthenia gravis, hsa_circ_0062400, hsa_circ_0002397, miR-338-3p, NRP1, therapeutic targets, autoimmune diseases

## Abstract

**Introduction:**

CircRNAs are implicated in various autoimmune diseases, such as Myasthenia Gravis (MG), yet their regulatory mechanisms remain poorly understood. This research investigated the regulatory network of circRNAs, miRNAs, and mRNAs in modulating MG progression.

**Materials and Methods:**

Three MG patients (male: female = 1:2) and three healthy controls were enrolled for microarray analysis. The “Limma” package was employed to identify the differentially expressed circRNAs (DECs). Target miRNAs of DECs were predicted *via* the CircInteractome database, while target genes of miRNAs were predicted utilizing miRWalk, miRTarbase, and TargetScan databases. Cytoscape software was applied to establish the circRNAs-miRNAs-mRNAs network. Thereafter, a dual-luciferase reporter assay was conducted to validate the targeted regulatory relationship between hsa_circ_0062400, hsa_circ_0002397, and miR-338-3p. The expression of *NRP1* was measured by qRT-PCR and Western blotting, and the cell viability of Jurkat cells was evaluated *via* CCK-8 assay. The levels of cytokines (IL-2, IL-6, IFN-γ, TNF-α) after *NRP1* knockout were detected by ELISA.

**Results:**

4 circRNAs, 2 miRNAs, and 11 target genes associated with MG pathogenesis were identified to construct a ceRNA regulatory network. *In-vitro* assays validated the targeted interactions between hsa_circ_0062400, hsa_circ_0002397, and miR-338-3p. miR-338-3p negatively regulated both protein and mRNA levels of *NRP1*. Further, silencing hsa_circ_0062400 or hsa_circ_0002397 markedly suppressed *NRP1* expression and Jurkat cell proliferation, which was reversed by miR-338-3p inhibitor. Moreover, *NRP1* affected the levels of cytokines in Jurkat cells.

**Discussion:**

The circRNAs were demonstrated to be closely associated with MG etiology, which was also supported by our current discovery that hsa_circ_0062400 and hsa_circ_0002397 regulated the level of *NRP1* by sponging miR-338-3p in MG. However, the roles of hsa_circ_0062400 and hsa_circ_0002397 in MG have been rarely reported, which requires further validation. Limitations of this study included a small, gender-imbalanced sample size for microarray analysis and incomplete verification of the ceRNA network. In addition, functional experiments were limited to Jurkat cells, and more *in-vivo* validation assays were lacking.

**Conclusion:**

Collectively, the present study revealed MG pathogenesis and also provided potential treatment targets for the disease.

## 1 INTRODUCTION

Myasthenia Gravis (MG) is a prevalent autoimmune disorder affecting the neuromuscular junction through the specific autoantibody [1]. The incidence of this disease ranges from 1.77 to 21.3 per million population, and the prevalence is between 15 million and 179 million [2]. The incidence rate of MG is 150 to 250 per million worldwide in China, with slightly higher prevalence in females than in males [3]. MG usually presents as fatigue fluctuation and muscle weakness, which may localize to ocular Muscles (oMG) or extend to other muscle groups (generalized MG, gMG) [4, 5]. Early and accurate diagnosis of MG remains a major clinical challenge due to its manifestations similar to conditions such as stroke, motor neuron illness, and inflammatory neuropathy [6]. Previous research has demonstrated that MG is primarily triggered by the dysfunction of immune tolerance, leading to the production of autoantibodies against the acetylcholine receptor in around 85% of patients, which subsequently initiates an aberrant immune response [7]. The Muscle-specific receptor tyrosine kinase (MuSK) or low-density lipoprotein receptor-related protein 4 (LRP4) may also be targets of autoimmune attack [8]. Based on the aforementioned characteristics, MG can be classified into subtypes, including early-oMG and late-oMG, seronegative MG, thymoma-associated MG, LRP4-positive MG, and MuSK-positive MG [9]. Although the cholinergic pathway and inflammatory factors play crucial roles in the initiation of MG and its progression, the underlying pathogenetic mechanism is still poorly understood [10]. The core pathological chain of MG involves two key links: first, the imbalance of autoimmune tolerance caused by abnormal activation of immune cells, and second, the impairment of neuromuscular signal transmission due to the destruction of neuromuscular junction structure. The cell polarity pathway is deeply involved in these two core links by regulating immune cell functions and the structural homeostasis of the neuromuscular junction. From the perspective of immune mechanisms, cell polarity controls cell motility, which is crucial for scanning peripheral tissues to detect pathogens and recruiting immune cells to infection sites [11]. Thus, further investigation into the pathogenesis of MG within the cell polarity pathway is essential for improving MG treatment.

As non-coding RNAs, circRNAs form a closed-loop configuration through specialized splicing modes [12]. CircRNAs play vital roles in cell proliferation [13], apoptosis [14], autoimmunity [15], and other biological processes. It is known that circRNAs, for instance, peripheral blood hsa-circRNAs, are highly conserved and stable, making them promising candidates for the detection and management of various diseases [16-18]. Circular RNA 5333-4 has been identified as a new diagnostic signature for MG [19]. MicroRNAs (miRNAs), endogenous non-coding small RNAs, function as regulators to suppress the expression of their mRNAs at the post-transcriptional level [20, 21]. Importantly, numerous studies have shown that circRNAs can act as the “sponge” for downstream miRNAs, thereby regulating diverse immune responses and immune-relevant disorders, including MG, *via* the competing endogenous RNA (ceRNA) mechanism [14]. For example, Lai *et al*. showed that overexpression of circ-FBL facilitates myogenic proliferation in MG through modulating the miR-133/PAX7 axis [22]. Despite these findings, the specific mechanisms of ceRNAs network involved in MG progression remained unclear.

We employed computational analysis to identify potential circRNAs, miRNAs, and target genes involved in MG pathogenesis, to establish a ceRNA regulatory network, and to screen the hub objects for further *in vitro* verification. This study addressed the current knowledge gap regarding the specific mechanisms through which circRNA-mediated regulatory networks contributed to MG development, with a particular focus on elucidating the association between the circRNA-miRNA-mRNA axis and the occurrence of MG, thereby providing a novel targeted regulatory pathway underlying the molecular mechanisms of MG. Based on computational predictions, we developed a circRNAs-mRNAs-ceRNAs regulatory network. Within this network, both hsa_circ_0062400 and hsa_circ_0002397 were predicted to form a regulatory relationship through targeting and binding to miR-338-3p, which could act on *NRP1*. We therefore specifically investigated whether hsa_circ_0062400 and hsa_circ_0002397 affected the expression of *NRP1* in Jurkat cells by regulating miR-338-3p. Against the backdrop of unclear ceRNA network mechanisms in MG progression, this study identifies key circRNAs from the cell polarity pathway, focusing on verifying the regulatory axis of hsa_circ_0062400/hsa_circ_0002397-miR-338-3p-NRP1, which fills the gap in understanding the role of these two circRNAs in MG and provides novel insights into the molecular mechanisms and therapeutic targets of MG.

## 2 MATERIALS AND METHODS

### 2.1 Sample Collection

Informed consents were signed by all the participants. Six samples, including 3 healthy controls and one male and two female patients with MG, were recruited for microarray analysis, with the age of the participants around 70 years old. All patients were newly diagnosed with generalized MG and had not received immunosuppressants, glucocorticoids, or cholinesterase inhibitors prior to the study. Healthy controls had no history of autoimmune diseases or neurological disorders, with normal liver and kidney function, and had not taken immunomodulators or hormonal drugs within the previous 3 months. Patients with other autoimmune or neurological diseases, severe systemic comorbidities, or prior use of immunosuppressants/immunomodulators were excluded. Individuals with recent infections or incomplete clinical data were also excluded.

The study was approved by the Ethics Committee of the Second Affiliated Hospital of Harbin Medical University (No. YJSKY2024-394). All the patients recruited into this study were positive for acetylcholine receptor autoantibodies and diagnosed with MG for the first time. The study period of this research is from May 2024 to October 2024.

### 2.2 Differentially Expressed CircRNAs (DECs) Analysis

Firstly, circRNA microarray scanning was performed on the above 6 samples. Expression profile data were analyzed employing the “Limma” R package to screen the DECs between the MG group and the control group [23]. The circRNAs with Fold Change (FC) ≥1.2 and *p* ≤0.05 were identified as the DECs, which were visualized in a heatmap.

### 2.3 Functional Enrichment Analysis

DECs were subjected to GO enrichment analysis by accessing the Metascape database (see https://metascape.org/gp/index.html#/main/step1) [24, 25]. The gene set related to the establishment or maintenance of cell polarity was collected from the Molecular Signatures Database (MSigDB), and the circRNAs associated with this pathway were obtained from circBase (https://www.circbase.org/). These circRNAs were then intersected with the DECs to identify the key circRNAs.

### 2.4 Screening of miRNAs

For miRNA screening, by accessing CircInteractome (https://circinteractome.nia.nih.gov/mirna_target_sites.html), all potential binding sites for miRNAs on the candidate circRNAs were predicted [26]. All resulting miRNAs were included in the subsequent analysis without additional screening or sorting.

### 2.5 Target Gene Prediction of miRNAs

Target genes of miRNAs were predicted by miRWalk, miRTarbase, and TargetScan databases. Specifically, for miRTarBase, only miRNA–mRNA interactions with experimental validation were retained; for miRWalk, the website's default parameters were used, and target genes with a predicted score ≥ 0.95 were selected; for TargetScan, all predicted target genes in the database were retained. Finally, the results from the three databases were intersected to obtain high-confidence candidate mRNAs.

### 2.6 Establishment of circRNAs-miRNAs-mRNAs ceRNA Network

A ceRNA network was established from the DECs by integrating predicted miRNAs-target genes and circRNAs-target miRNAs related to MG pathogenesis. The network was visualized by the Cytoscape 3.9.1 software [27].

### 2.7 Culture and Transfection of Cells

Roswell Park Memorial Institute 1640 medium (11875093, Gibco, Waltham, MA, USA) with 10% fetal bovine serum and 1% penicillin/streptomycin was employed to culture human T-lymphocytic leukemia cell line Jurkat (C5187, BDBio, Hangzhou, China) at 37°C with 5% CO_2_ in an incubator. The Jurkat cell line, derived from a T-cell lymphoblastic leukemia case, has been widely used as a model T-cell line in biomedical research [28]. Previously, numerous studies on MG have also utilized Jurkat cells [29].

In this study, Jurkat cells were transfected using Lipofectamine 3000 reagent (L3000150, Invitrogen, Carlsbad, CA, USA) according to the manufacturer's instructions. The pcDNA-3.1 (+) vectors (V79020, Invitrogen, USA) were utilized for overexpressing hsa_circ_0062400 (oe-hsa_circ_0062400) and hsa_circ_0002397 (oe-hsa_circ_0002397), while the empty vector served as a negative control. The miR-338-3p inhibitor and mimic, along with relevant controls, were ordered from GenePharma (Shanghai, China). Moreover, the specific siRNA to hsa_circ_0062400 (si-hsa_circ_0062400, target sequence: 5’-CGGAAAGCATGGAAATAGGAATT-3’), hsa_circ_0002397 (si-hsa_circ_0002397, target sequence: 5’- TTGCAAAGAGATTTGAAAAGAAC-3’), *NRP1* (si-*NRP1*#1, 5’-CAGAAGAATGGTACAAATCCAAG-3’; si-*NRP1*#2, 5’-CCCTTAAAGGAACCAATGAGTCC-3’), and the negative control (si-NC) were ordered from Sangon Biotech (Shanghai, China).

### 2.8 Dual-luciferase Reporter Assay

Utilizing Lipofectamine 3000 reagent (L3000150, Invitrogen, USA), Jurkat cells were co-transfected with the mutant type or wild-type of miR-338-3p (miR-338-3p wt/mut) vectors, hsa-NC, and oe-hsa_circ_0062400, oe-hsa_circ_0002397. After 48 h, the Dual-Luciferase Reporter Assay System (E1910, Promega, Madison, WI, USA) was used to measure relative luciferase activity according to the protocol. The activity was normalized to that of Renilla [30].

### 2.9 Cytokine Detection

The si-*NRP1*#1 and si-*NRP1*#2 were transfected using Lipofectamine 3000 reagent (L3000150, Invitrogen, Carlsbad, CA, USA) into Jurkat cells following the protocols, with a negative control group (si-NC) set up in parallel.

The Jurkat cells with higher *NRP1* transfection efficiency were selected for subsequent analysis. Cell culture supernatants from both the si-*NRP1* and si-NC groups were collected and centrifuged at 1000-1500 rpm for 5 to 10 min to remove cellular debris and other impurities. The resulting supernatants were subjected to ELISA assays using the Human IL-2 ELISA Kit (PI580, Beyotime, Shanghai, China), Human IL-6 ELISA Kit (PI330, Beyotime), Human Interferon (IFN)-γ ELISA Kit (PI511, Beyotime), and Human Tumor Necrosis Factor (TNF)-α ELISA Kit (PT518, Beyotime) to determine the concentrations of IL-2, IL-6, IFN-γ, and TNF-α, respectively.

### 2.10 QRT-PCR

Following the specification, the total RNA was separated from the transfected Jurkat cells employing TRIzol reagent (15596026CN, Invitrogen, USA) and then reverse-transcribed into cDNA utilizing the RevertAid First Strand cDNA Synthesis Kit (K1621, Thermo Fisher Scientific, Waltham, MA, USA). Afterwards, PCR amplification was conducted using the PowerTrack™ SYBR Green Master Mix (A46110, Thermo Fisher Scientific, USA). The method of 2^-ΔΔct^ was applied to calculate the relative mRNA expression of *NRP1*, with *GAPDH* as a housekeeping gene [31]. The primers employed in this research were: *NRP1*-F 5’-AACAACGGCTCGGACTGGAAGA-3’, *NRP1*-R 5’-GGTAGATCCTGATGAATCGCGTG-3’; *GAPDH*-F 5’-GTCTCCTCTGACTTCAACAGCG-3’, *GAPDH*-R 5’-ACCACCCTGTTGCTGTAGCCAA-3’.

### 2.11 Western Blotting

Total protein was first collected from the transfected Jurkat cells utilizing radio-immunoprecipitation lysis buffer (GK10023, GlpBio, Montclair, CA, USA) and then detected with the BCA protein assay kit (K813-5000, Amyjet, Wuhan, China) [32]. Then, the proteins were separated by SDS-PAGE and transferred onto PVDF membrane (LC2007, Invitrogen, USA). Subsequently, the film was blocked with 5% defat milk for 2 h. Next, the membrane was first incubated with primary antibodies against NRP1 (ab81321, 1:1000, Abcam) and internal reference GAPDH (ab8245, 1:1000, Abcam) at 4°C overnight and then with secondary antibodies HRP-conjugated goat anti-rabbit IgG (ab97051, 1:5000, Abcam) and anti-mouse IgG (ab97023, 1:5000, Abcam) at ambient temperature for 1.5 h. Ultimately, an ECL system (WBULS0100, Millipore, Merck, Germany) was used to visualize the bands according to the specifications.

### 2.12 CCK-8 Assay

The viability of Jurkat cells was detected by CCK-8 assay. Briefly, the transfected Jurkat cells (5000 cells/well) were planted into a 96-well plate and incubated at 37°C for 0, 6, and 12 h. Afterwards, these cells were processed with 10 μL CCK-8 solution (CA1210, Solarbio, Beijing, China) for another 2 h. The Varioskan ALF Multimode Microplate Reader (VA000010C, Thermo Fisher Scientific, USA) was employed to measure cell viability at the absorbance of 450 nm.

### 2.13 Statistical Analysis

The data were analyzed in R software (version 4.0.1) or GraphPad Prism software (version 8.0.2). All the experiments were conducted in independent triplicates, and data were shown as mean ± SD. All experiments were performed with three independent replicates (n = 3). Comparisons among different groups were conducted employing the one-way or two-way ANOVA method. Statistically significant difference was defined at *p* < 0.05.**** means *p* < 0.0001, *** means *p* < 0.001, ** means *p* < 0.01

## 3 RESULTS

### 3.1 Screening for DECs and Functional Enrichment Analyses

**Fig. (1):**
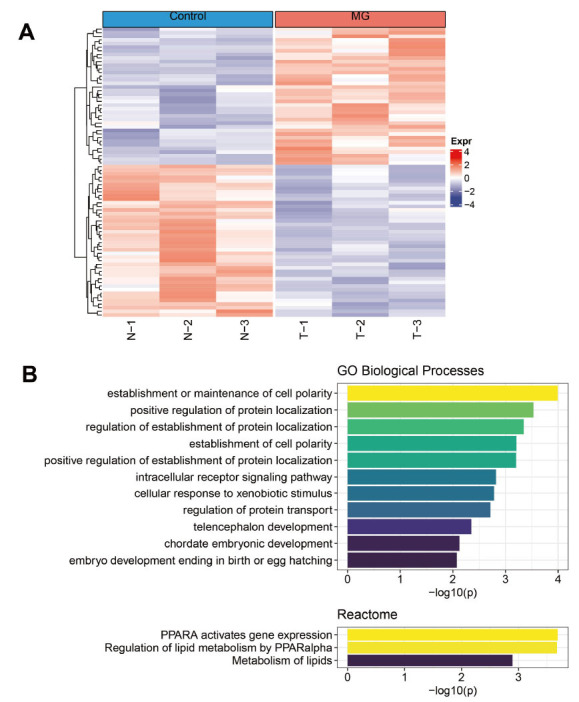
Screening of DECs and functional enrichment analyses. (**A**) Heatmap of DECs between MG patients and healthy controls. This figure shows the expression profiles of differentially expressed circRNAs between the MG group (T-1, T-2, T-3) and the healthy control group (N-1, N-2, N-3). Each column indicates a sample, and each row indicates a circRNA. The expressions were normalized by Z-score and indicated by a color gradient: blue and red represent low and high expression, respectively (**B**) GO and Reactome enrichment analysis of differentially upregulated circRNAs.

Limma R package screened 38 upregulated circRNAs and 42 downregulated circRNAs between 3 MG patients and 3 healthy controls (Fig. **1A**). GO analysis of the differentially upregulated circRNAs was performed using the Metascape website. These circRNAs were mainly involved in the establishment or maintenance of cell polarity, PPARA activates gene expression, positive regulation of protein localization, and regulation of the establishment of protein localization (Fig. **1B**). As the establishment or maintenance of cell polarity is the most enriched pathway, it is involved in the establishment or maintenance of cell polarity, which played a crucial part in the occurrence of MG. The stability of the neuromuscular junction depends on normal cell polarity, and its imbalance might lead to impaired neuromuscular signal transmission and MG [33]. Therefore, the establishment or maintenance of the cell polarity term was selected for subsequent analysis.

### 3.2 Development of a circRNAs-miRNAs Regulatory Network

**Fig. (2):**
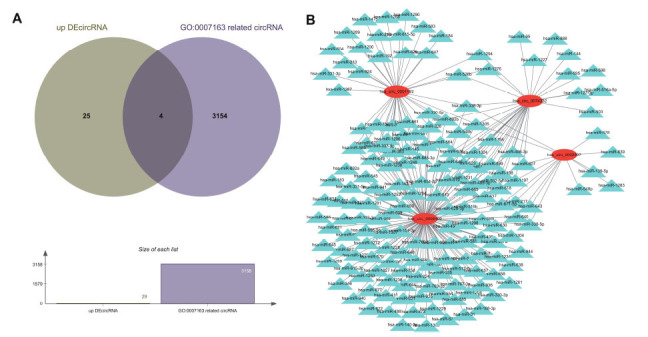
Construction of the circRNAs-miRNAs regulatory network. (**A**) Screening of crucial circRNAs; (**B**) circRNAs-miRNAs regulatory network, the red ellipse represents circRNAs and the blue triangle represents miRNAs.

The gene set associated with the establishment or maintenance of cell polarity (GO:0007163) was downloaded from the MSigDB. Then, using circBase, 3158 circRNAs related to this pathway were retrieved and intersected with the upregulated DECs, resulting in the identification of 4 critical circRNAs (hsa_circ_0062400, hsa_circ_0074332, hsa_circ_0004183, hsa_circ_0002397) (Fig. **2A**). The target miRNAs of these 4 circRNAs were predicted *via* the CircInteractome database. A total of 175 miRNAs and 228 regulatory pairs were identified to construct a circRNAs-miRNAs network using Cytoscape 3.9.1 software (Fig. **2B**).

### 3.3 Target Gene Prediction of miRNAs

**Fig. (3):**
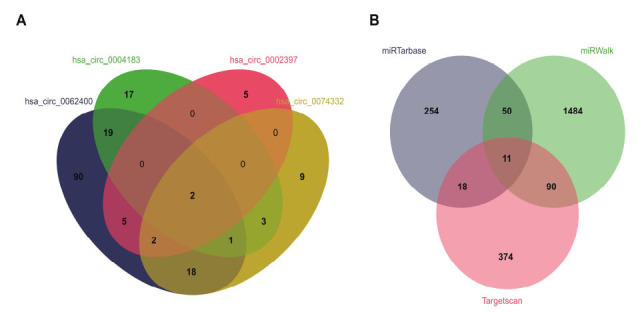
Prediction of miRNA target genes. (**A**) Prediction of the common miRNAs of the 4 circRNAs (hsa_circ_0062400, hsa_circ_0004183, hsa_circ_0002397 and hsa_circ_0074332). (**B**) Prediction of the target mRNAs of the miR-338-3p based on the databases of miRTarbase, TargetScan and miRWalk.

The Venn diagram showed that hsa-miR-1305 and hsa-miR-338-3p were common to all the 4 key circRNAs (Fig. **3A**). Subsequently, the target genes of hsa-miR-1305 databases. A total of 11 common target genes for hsa-miR-338-3p were identified by screening with miRWalk, miRTarBase, and TargetScan, and then intersecting the results from these three databases (Fig. **3B**).

### 3.4 Establishment of a CircRNAs-mRNAs-ceRNAs Network

**Fig. (4):**
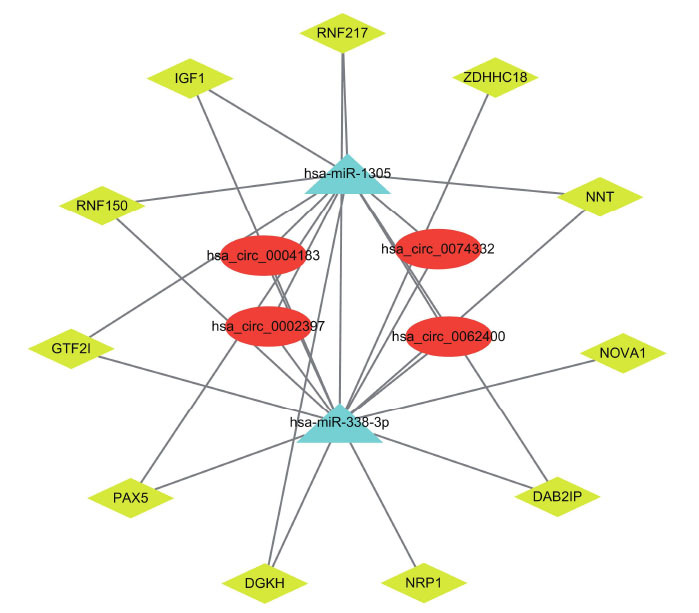
Construction of a ceRNA network consisting of circRNAs, miRNAs, and mRNAs. **Note:** The red ellipse indicates circRNAs, the blue triangle indicates miRNAs, and the yellow diamond indicates mRNAs.

Subsequently, the 2 miRNAs (hsa-miR-1305, hsa-miR-338-3p), 4 circRNAs (hsa_circ_0062400, hsa_circ_0074332, hsa_circ_0004183, hsa_circ_0002397), and 11 target genes (*NNT*, *NOVA1*, *DAB2IP*, *NRP1*, *DGKH*, *PAX5*, *GTF2I*, *RNF150*, *IGF1*, *RNF217*, *ZDHHC18*) potentially associated with MG pathogenesis were integrated to construct a circRNAs-miRNAs-mRNAs regulatory network (Fig. **4**). Among these, *NRP1* has been shown to be highly correlated with the development of systemic autoimmune diseases [34]. Therefore, in our circRNAs-miRNAs-mRNAs regulatory network, *NRP1* was found to be regulated by the upstream miR-338-3p, which was predicted to be the potential target of hsa_circ_0062400 and hsa_circ_0002397 (Fig. **4**). Therefore, subsequent research focused specifically on a part of this subnetwork, with emphasis on investigating the mechanism by which hsa_circ_0062400 and hsa_circ_0002397 target miR-338-3p to regulate *NRP1* in MG development.

### 3.5 Hsa_circ_0062400 and hsa_circ_0002397 Targeted miR-338-3p

**Fig. (5):**
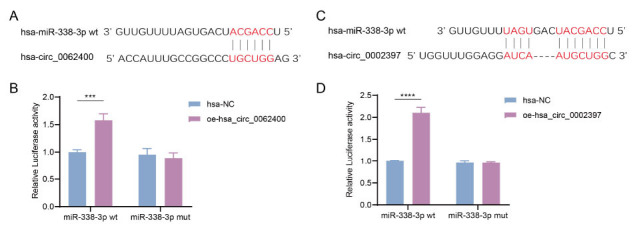
Hsa_circ_0062400 and hsa_circ_0002397 interacts with miR-338-3p. (**A**) The binding sites of miR-338-3p wt with hsa_circ_0062400; (**B**) Dual-luciferase reporter assay validated the direct interaction between miR-338-3p and hsa_circ_0062400 in Jurkat cells; (**C**) The binding sites of miR-338-3p wt with hsa_circ_0002397; (**D**) The direct interaction between miR-338-3p and hsa_circ_0002397 in Jurkat cells was verified through dual-luciferase reporter assay. Data were shown as mean ± SD. And *** means *p* < 0.001, **** means *p* < 0.0001.

The targeted regulatory correlation between hsa_circ_0062400, hsa_circ_0002397, and miR-338-3p was validated in Jurkat cells using a dual-luciferase reporter assay. The binding sites of miR-338-3p wt with hsa_circ_0062400 were displayed in Fig. (**5A**). Overexpressing hsa_circ_0062400 markedly elevated the relative luciferase activity of miR-338-3p wt, but this effect was abolished by miR-338-3p mut (Fig. **5B**, *p* < 0.05). Similarly, binding sites were identified between miR-338-3p wt and hsa_circ_0002397 (Fig. **5C**). Overexpression of hsa_circ_0002397 did not significantly enhance the relative luciferase activity of miR-338-3p wt, but miR-338-3p mut attenuated the effect (Fig. **5D**, *p* < 0.05). Hence, there may be a targeted interaction between miR-338-3p, hsa_circ_0062400, and hsa_circ_0002397.

### 3.6 Negative Regulation of *NRP1* by miR-338-3p

**Fig. (6):**
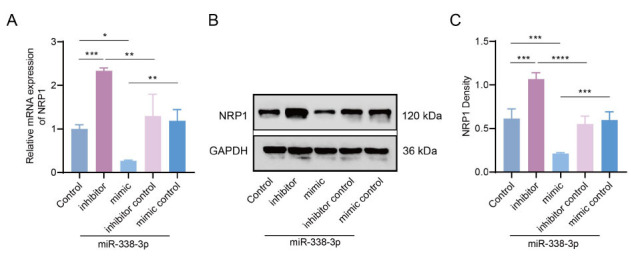
miR-338-3p negatively regulates the expression of *NRP1*. (**A**) qRT-PCR detection of the relative mRNA expression of *NRP1* in Jurkat cells transfected with negative control, miR-338-3p inhibitor or miR-338-3p mimic; (**B-C**) Western blotting examining the protein level of NRP1 in Jurkat cells after transfection with negative control, miR-338-3p inhibitor or miR-338-3p mimic. Data were shown as mean ± SD. Among them, the “inhibitor” group refers to the Jurkat cell group transfected with the miR-338-3p inhibitor, and the “mimic” group refers to the Jurkat cell group transfected with the miR-338-3p mimic. And * means *p* < 0.05, ** means *p* < 0.01, *** means *p* < 0.001, **** means *p* < 0.0001.

QRT-PCR and Western blotting showed that protein and mRNA expressions of *NRP1* were notably elevated in Jurkat cells transfected with miR-338-3p inhibitor, while *NRP1* expression was observably downregulated in the miR-338-3p mimic group (Figs. **6A**-**C**, *p* < 0.05). These data revealed that the expression of *NRP1* was negatively regulated by miR-338-3p.

### 3.7 Hsa_circ_0062400 and hsa_circ_0002397 Targeted miR-338-3p to Regulate the Level of *NRP1*

**Fig. (7):**
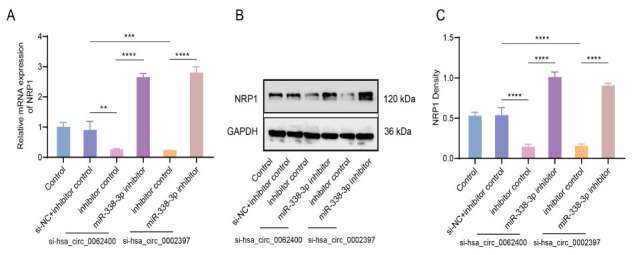
Hsa_circ_0062400 and hsa_circ_0002397 target miR-338-3p to regulate *NRP1* expression. (**A**) The relative mRNA expression of *NRP1* in transfected Jurkat cells measured by qRT-PCR assay; (**B-C**) The protein expression of NRP1 in transfected Jurkat cells by Western blotting assay. Data were shown as mean ± SD. Among them, the “inhibitor” group refers to the Jurkat cell group transfected with the miR-338-3p inhibitor, and the “mimic” group refers to the Jurkat cell group transfected with the miR-338-3p mimic. And ** means *p* < 0.01, *** means *p* < 0.001, **** means *p* < 0.0001.

Whether hsa_circ_0062400 and hsa_circ_0002397 regulated *NRP1* expression in Jurkat cells through targeting miR-338-3p was validated. The mRNA and protein levels of NRP1 in Jurkat cells were markedly inhibited after silencing hsa_circ_0062400 or hsa_circ_0002397, and this inhibition was reversed by co-transfection with the miR-338-3p inhibitor (Figs. **7A**-**C**, *p* < 0.05). Hsa_circ_0062400 and hsa_circ_0002397 targeted miR-338-3p to regulate the level of *NRP1,* as shown by qRT-PCR and Western blotting assays.

### 3.8 Hsa_circ_0062400 and hsa_circ_0002397 Modulated Jurkat Cell Proliferation *via* Targeting miR-338-3p

**Fig. (8):**
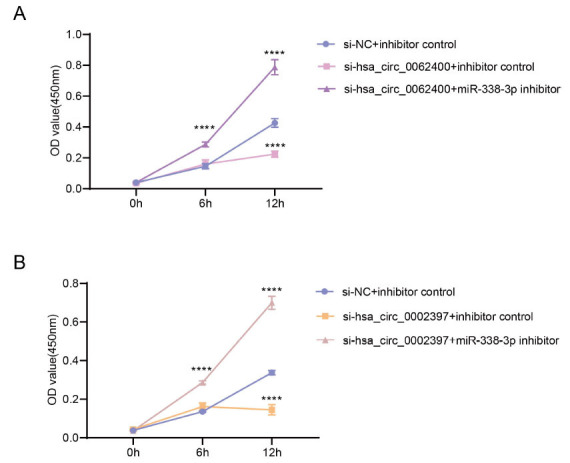
Hsa_circ_0062400 and hsa_circ_0002397 modulate Jurkat cell proliferation *via* targeting miR-338-3p. (**A**) CCK-8 method assessing cell proliferation of Jurkat cells transfected with si-NC+inhibitor control, si-hsa_circ_0062400+inhibitor control, or si-hsa_circ_0062400+miR-338-3p inhibitor; (**B**) Cell proliferation evaluated by CCK-8 assay in Jurkat cells transfected with si-NC+inhibitor control, si-hsa_circ_0002397+inhibitor control, or si-hsa_circ_0002397+miR-338-3p inhibitor. Data were shown as the mean ± SD. And **** means *p* < 0.0001.

CCK-8 assay showed that silencing hsa_circ_0062400 or hsa_circ_0002397 remarkably suppressed Jurkat cell viability, whereas the miR-338-3p inhibitor reversed the suppression of cell viability (Figs. **8A**-**B**, *p* < 0.05). Collectively, hsa_circ_0062400 and hsa_circ_0002397 could modulate Jurkat cell proliferation *via* targeting miR-338-3p, which might be linked to the development of MG.

### 3.9 *NRP1* Affected the Expression of Cytokines in Jurkat Cells

**Fig. (9):**
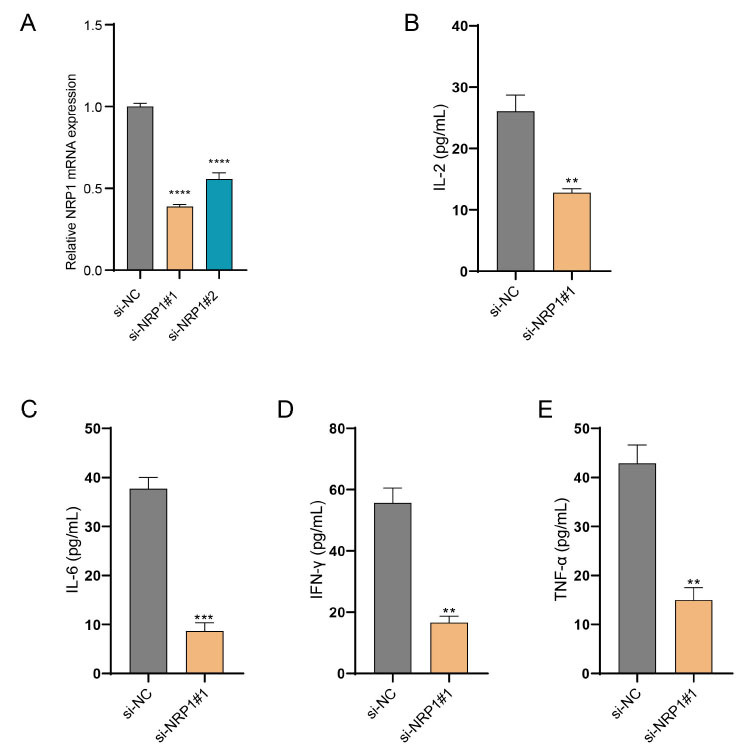
Expression of cytokines after *NRP1* knockout. (**A**) Validation of *NRP1* knockout efficiency. (**B**) The expression of IL-2 was significantly decreased after *NRP1* knockout. (**C**) The expression of IL-6 was significantly decreased after *NRP1* knockout. (**D**) The expression of IFN-γ was significantly decreased after *NRP1* knockout. (**E**) The expression of TNF-α was significantly decreased after *NRP1* knockout. And ** means *p* < 0.01, *** means *p* < 0.001, **** means *p* < 0.0001.

To further verify the function of *NRP1* in Jurkat cells, we knocked down *NRP1* and selected si-*NRP1*#1 with higher knockdown efficiency for subsequent cytokine detection (Fig. **9A**, *p* < 0.05). It was observed that compared to the si-NC group, the expressions of IL-2, IL-6, IFN-γ, and TNF-α in the si-*NRP1*#1 group were significantly inhibited (Figs. **9B**-**E**, * p* < 0.05).

## 4 DISCUSSION

MG is a common autoimmune disease and a chronic neuromuscular disorder that significantly affects patients' quality of life [35]. Numerous studies have demonstrated that disruption of the ceRNA network could induce tumorigenesis, such as colorectal cancer [36], and immune-relevant diseases, including MG [37]. This study constructed a ceRNA network consisting of 4 circRNAs, 2 miRNAs, and 11 target genes. Among these, hsa_circ_0062400 and hsa_circ_0002397 were shown to regulate *NRP1* expression and Jurkat cell proliferation by targeting miR-338-3p, which may be closely implicated in the development of MG. These findings may enhance the understanding of MG pathogenesis and provide new insights for therapeutic strategies.

Recently, circRNAs have been found to exert their biological function by acting as ceRNAs for miRNAs to regulate downstream mRNA expression, playing important roles in modulating disease progression [38]. For instance, previous research revealed that circVMA21 regulates the expression of *NRP1* in acute kidney injury by sponging miR-199a-5p [39]. Specifically, through adsorbing miRNAs like a “sponge”, circRNAs prevent miRNAs from binding to their target mRNAs, thereby alleviating miRNA-mediated inhibition on target genes and ultimately influencing gene expression and cellular functions. Zhou *et al*. demonstrated that circ_0006089 promotes gastric cancer progression *via* targeting the miR-217/NRP1 axis [40]. Lai *et al*. reported that upregulation of circ-FBL enhances myogenic proliferation in MG through sponging miR-133/PAX7 [22]. Furthermore, studies have shown that the m^6^A methylation levels of hsa_circ_0084735 and hsa_circ_0025731 were downregulated in patients with MG [41]. This study confirmed that hsa_circ_0062400 and hsa_circ_0002397 directly bound to miR-338-3p. Meanwhile, miR-338-3p negatively regulated *NRP1* expression. Previous studies have also demonstrated that miR-338-3p expression promotes antigen-specific Th17 cell responses [42]. Given that anti-inflammatory regulatory T cells (Tregs) and pro-inflammatory T helper 17 (Th17) cells exert functionally antagonistic effects, the immune imbalance between these two cell subsets is recognized as one of the pathogenic factors contributing to MG [43]. This regulatory axis may be implicated in the mechanism by which miR-338-3p influences MG pathogenesis. In addition to the *NRP1* pathway, studies have also found that circ_0076490 regulates the expression of MAPK1 through interacting with miR-144-3p to influence the development of MG [44]. However, the roles of hsa_circ_0062400 and hsa_circ_0002397 in MG remained largely unexplored and required further validation.

The study showed that miR-338-3p, located at chromosome 17q25, is transcribed from intron 8 of the apoptosis-relevant tyrosine kinase gene [45]. Research indicates that miR-338-3p can inhibit the development of multiple cancers, including ovarian cancer [46] and gastric cancer [47]. Notably, miR-338-3p plays an imperative role in facilitating cell apoptosis, neuronal differentiation, and neurite elongation [48, 49]. Previous studies have also demonstrated that the lncRNA *MALAT-1* could serve as a ceRNA to modulate the level of *MSL2* *via* targeting miR-338-3p in MG [50]. Additionally, our CCK-8 assay revealed that silencing hsa_circ_0062400 or hsa_circ_0002397 markedly inhibited Jurkat cell viability, whereas the miR-338-3p inhibitor reversed this downregulation in *NRP1* expression. Collectively, these findings confirmed that the hsa_circ_0062400/ and hsa_circ_0002397/miR-338-3p/*NRP1* axis was linked to MG progression.

Neuropilin 1 (*NRP1*), a transmembrane glycoprotein expressed on nerve fiber axons, is involved in angiogenesis, tumorigenesis, nervous system development, and immune function [51]. It has been shown that abnormally upregulated *NRP1* in many tumors, such as bladder cancer [52] and HNSCC [53], promotes disease progression. Importantly, aberrantly high-expressed *NRP1* can also accelerate the development of systemic autoimmune diseases [34]. Studies found that *NRP1* is closely associated with the regulation of CD8^+^ T cells and has been identified as a marker for thymically derived murine regulatory T (Treg) cells [54]. *NRP1* may be involved in neuromuscular junction function in MG, particularly in neuro-immune interactions. A previous study reported that overexpression of NRP1 in motoneurons facilitates the destruction of neuromuscular junctions in amyotrophic lateral sclerosis [55]. Furthermore, the present study found that NRP1 knockout affected MG-related phenotypes, manifesting as alterations in cytokine secretion. Specifically, after *NRP1* knockout, the expressions of TNF-α, IFN-γ, IL-2, and IL-6 were all markedly inhibited, suggesting that *NRP1* may be implicated in the pathological processes of MG through regulating the expression of these cytokines. Mechanistically, IL-2, a T-cell growth factor, can promote the differentiation and proliferation of effector T cells and play a role in enhancing immune responses in the immune system. Previous studies confirmed that the serum level of IL-2 in MG patients is significantly elevated [56]. Meanwhile, TNF-α and IFN-γ can stimulate B cells to produce pathogenic antibodies, which is also one of the important mechanisms implicated in the pathogenesis of MG [51]. In addition, among the two main functional subsets of helper T cells (Th cells), Th1 cells secrete pro-inflammatory cytokines such as IFN-γ and IL-2, while Th2 cells secrete cytokines including IL-4 and IL-6. The study also showed that IL-6 can further stimulate B cell growth, differentiation, and antibody production [57]. These cytokines may contribute to the pathogenesis of Myasthenia Gravis (MG) by disrupting T cell homeostasis. For instance, IL-2 promotes the proliferation of helper T cells, while TNF-α accelerates the apoptosis of regulatory T cells, thereby further amplifying T cell imbalance [58]. Studies have shown that *NRP1* can alleviate inflammatory responses by regulating the PI3K/AKT/mTOR signaling pathway [59] and modulate T cell function through co-expression with LAP-TGF-β1 [60]. These processes may be associated with the mechanism by which NRP1 influences cytokine expression in MG. In summary, *NRP1* may contribute to MG pathogenesis through influencing the expression of the aforementioned key cytokines and immune regulatory imbalance or directly participating in the structural and functional damage of neuromuscular junctions, thereby emerging as a potential key molecule linking immune abnormalities and neuromuscular transmission disorders.

Nevertheless, some limitations in this work should be noted. First, the microarray analysis involved a limited sample size with an imbalanced gender ratio, affecting the representativeness of the findings. To improve the reliability and applicability of our conclusions, future studies will validate these results in larger and more diverse cohorts to include different subtypes of MG. Second, the established ceRNA network consisted of 4 circRNAs, 2 miRNAs, and 11 target genes, but only the mechanism of hsa_circ_0062400/ and hsa_circ_0002397/miR-338-3p/NRP1 axis in MG progression was verified *in vitro* using Jurkat cells. Whether the other circRNAs/miRNAs/mRNAs were also involved in MG pathogenesis remained unclear. Finally, as for functional verification, we only used Jurkat cells to validate the interaction of circRNAs-miRNAs-mRNAs. Considering that the core pathological site of myasthenia gravis is at the neuromuscular junction and the complex involvement of immune cells, subsequent studies will include more cell lines that can simulate the characteristics of the neuromuscular junction and primary T cells to more accurately reflect *in vivo* physiological and pathological conditions and enhance the clinical transformation value of the research results. For instance, establish a neuromuscular co-culture model to observe its effects on neuromuscular junction formation and function, thereby bridging T cell immunoregulation with neuromuscular junction impairment. Additionally, validate using *in vivo* animal models to assess changes in neuromuscular junction structure and myasthenic symptoms in rats, thereby comprehensively verifying its regulatory role.

## CONCLUSION

To conclude, a circRNAs-miRNAs-mRNAs ceRNA network associated with MG pathogenesis was established through computational analysis. Further, *in vitro* assays verified that hsa_circ_0062400 and hsa_circ_0002397 modulated *NRP1* expression and Jurkat cell proliferation through sponging miR-338-3p. This study revealed the pathogenesis of MG and its treatment.

## Data Availability

Raw data containing donor-related information cannot be publicly shared. De-identified processed data and non-privacy-related raw data are available from the corresponding author upon reasonable request and with ethical approval.

## LIST OF ABBREVIATIONS

CCK-8: Cell Counting Kit-8
ceRNA: Competing Endogenous RNA
circRNA: Circular RNA
DEC: Differentially Expressed circRNA
FC: Fold Change
GO: Gene Ontology
MG: Myasthenia Gravis
miRNA: MicroRNA
mRNA: Messenger RNA
MSigDB: Molecular Signatures Database
NRP1: Neuropilin 1
qRT-PCR: Quantitative Real-Time PCR
siRNA: Small Interfering RNA
Treg: Regulatory T
Th Cell: Helper T Cell
IFN: Interferon
TNF-α: Tumor Necrosis Factor-α
